# The landscape of small nucleolar RNA expression in multiple myeloma is determined by cytogenetic alterations

**DOI:** 10.1038/s41375-023-02060-2

**Published:** 2023-10-16

**Authors:** Duoduo Zhao, Christian Rohde, Stefanie Göllner, Fengbiao Zhou, Cornelius Pauli, Maximilian Felix Blank, Rafael Zinz, Anna Luise Grab, Alexandra M. Poos, Lukas John, Stefanie Huhn, Marc-Steffen Raab, Carsten Müller-Tidow, Niels Weinhold

**Affiliations:** 1 Department of Internal Medicine V, Heidelberg University Hospital, Heidelberg, Germany; 2 Bone Marrow Transplantation Center, the First Affiliated Hospital, Zhejiang University School of Medicine, Hangzhou, China; 3 Molecular Medicine Partnership Unit (MMPU), European Molecular Biology Laboratory (EMBL), Heidelberg, Germany; 4 Division of Mechanisms Regulation Gene Expression, German Cancer Research Center (DKFZ), Heidelberg, Germany; 5 Division Proteomics of Stem Cells and Cancer, German Cancer Research Center (DKFZ), Heidelberg, Germany; 6 Clinical Cooperation Unit (CCU) Molecular Hematology/Oncology, German Cancer Research Center (DKFZ), Heidelberg, Germany

**Keywords:** Myeloma, Myeloma

## To the Editor:

Multiple myeloma (MM) is a plasma cell malignancy that accounts for approximately 10% of all hematologic malignancies [1]. Poor prognosis in MM is associated with chromosomal aberrations such as gain(1q), del(17p) and translocation t(4;14) [2]. However, the molecular mechanisms by which adverse cytogenetics impact on the course of the disease remains incompletely understood. In a recent study, the small nucleolar RNA (snoRNA) SCARNA22 was found to be overexpressed in MM patients with t(4;14) and was associated with reduced survival [3]. The snoRNAs are non-coding RNAs of 60–300 nucleotides [4], which can be divided into two classes: the C/D box (SNORDs), which guide 2′-O-methylation, and the H/ACA box snoRNAs (SNORAs), which direct pseudouridylation [5], both playing a regulatory role in ribosome biogenesis.

Emerging evidence suggests that snoRNAs contribute to oncogenesis in multiple cancers, *e.g*. acute myeloid leukemia (AML) and non-small cell lung cancer (NSCLC) [3, 6, 7]. SnoRNAs play a key role in the modification of ribosomal RNA, thus snoRNAs may exert important functions in MM, especially since immunoglobulin production requires intensive ribosome-dependent protein synthesis. However, the role of snoRNAs in MM has not been investigated thoroughly yet. Here, we combined mRNAseq, small RNAseq and ribosome methylation sequencing to investigate snoRNA expression patterns in MM and their association with different chromosomal aberrations. We identified snoRNAs dysregulated in molecular subgroups, with SNORD78 being highly expressed in gain(1q) patients and associated with poor prognosis. Our study shows that the expression of particular snoRNAs and methylation of specific snoRNA-guided rRNA sites are associated with specific chromosome gains, which are common elements in MM.

Previous profiling of small RNAs in MM has mainly focused on microRNAs and approaches are confined to PCR- or array-based techniques [8, 9]. Using NGS, we profiled the expression of snoRNAs in CD138-enriched MM cells from 71 newly diagnosed MM (NDMM) and four relapsed/refractory MM patients (Supplementary Table S1) as well as CD19^+^ B cells from three healthy donors. The detailed methodology and analysis workflow are provided in the “Supplementary Methods”.

We identified 625 full-length snoRNAs. Hierarchical clustering of the top100 most variably expressed snoRNAs revealed three distinct clusters which were associated with chromosomal abnormalities (Fig. S1A). Cluster 1 was enriched for patients with a hyperdiploid (HD) karyotype, while patients with IgH translocation were concentrated in cluster 2, and patients with gain(1q) in cluster 3. Of note, 74% of the top 100 most variably expressed snoRNAs guide 2′-O-methylation. Compared to CDl9^+^ B cells, myeloma cells displayed enrichment of SNORDs guiding 2′-O-methylation. This might be due to the increased activity of ribosomes in MM, as it necessitates the synthesis of a large amount of proteins. Therefore, we performed RiboMethSeq to investigate the RNA methylation status at all known sites in 26 NDMM samples (Fig. S1B). Overall, most known rRNA modification sites were highly methylated with little variation across samples. In addition, there were 39 sites with considerable intra-patient heterogeneity, so-called dynamic sites (variability in methylation score >0.02, shown in Fig. S1B). Such variability may indicate differences in translational preferences [6]. Recently, Zhou et al. demonstrated the dynamic rRNA 2′-O-methylation pattern in AML patients and its abundance associated with leukemia stem cell (LSC) activity by promoting the translation of proteins crucial for LSC [6].

Next, we in-depth analyzed the association between snoRNA expression and cytogenetic aberrations. We commonly observed that a set of dysregulated snoRNAs was enriched in a specific region in the altered chromosome. Directly comparing HD and non-HD patients revealed 50 significantly differentially expressed snoRNAs (Fig. S2A). The majority of these (82%) constituted SNORD115 family members on 15q11 (Fig. S2A, B) and were overexpressed in HD MM (Fig. S2C). Moreover, comparing the total normalized counts of SNORD115 family, the expression level of SNORD115 family members was increased in HD patients without gain(1q), compared to patients harboring an HD karyotype and gain(1q) simultaneously (Fig. S2D, t test, *P* = 0.001). Although the SNORD115 expression level was also associated with the copy number of chromosome 15 (Fig. S3A), all 37 HD patients carried multiple trisomies, with 34 of them having trisomy 15. Hence, it was challenging to establish a definitive association between the overexpression of SNORD115 and trisomy 15 (Fig. S3B, C).

We also investigated snoRNAs associated with the presence of distinct IgH translocations. High expression levels of SCARNA22 distinguished cases with t(4;14), which is consistent with prior studies [3] (Fig. S2E). The heatmap of differentially expressed snoRNAs reveals specific patterns of snoRNAs in patients with t(4;14) (Fig. S4). Compared to patients without t(4;14), certain H/ACA box snoRNAs targeting pseudouridylation, such as SNORA20, SNORA24 and SNORA14B, are highly expressed in patients carrying t(4;14). Patients with t(11;14) also clustered separately in differentially expressed snoRNAs (Fig. S5). C/D box snoRNAs are highly expressed in these patients, while H/ACA box snoRNAs exhibit low expression.

**Fig. 1:**
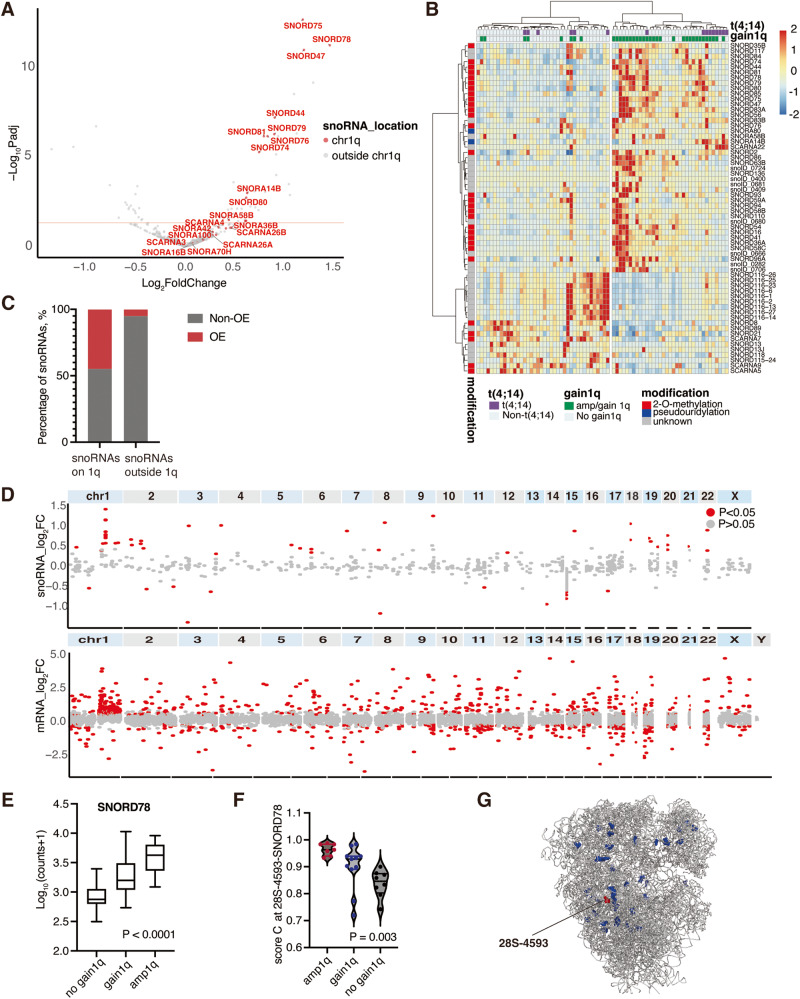
Patients with 1q gain express higher levels of snoRNAs expressed from 1q25 region. **A** Volcano plot of differentially expressed snoRNAs in gain1q *vs* non-gain1q patients. Scattered points represent individual snoRNAs: *red*-snoRNAs on chr1q, *grey*-snoRNAs outside chr1q. Red solid lines represents *Padj* < 0.05. **B** Heatmap of significant differentially expressed snoRNAs (*Padj* < 0.05). Most patients with gain1q were clustered together. A subset of snoRNAs located on chr1q was identified. **C** Most overexpressed snoRNAs were located on chr1q in patients with gain/amp1q. Among 29 snoRNAs on 1q, 13 of them (44.8%) were highly expressed while only 5.0% of snoRNAs outside chr1q were overexpressed (*P* < 0.001). **D** Distribution of snoRNAs and mRNAs on each chromosome. Each dot represents a single snoRNA or mRNA. Log_2_FC was calculated from 36 cases with gain/amp1q vs 39 non-gain1q patients. The red dot indicates significant ones. **E** The expression level of SNORD78 across myeloma patients. *Gain* indicates 3 copies of 1q21, *amp* indicates ≥ 4 copies of 1q21. **F** Methylation level of ribosome 28S-4593 site guided by SNORD78. **G** Distribution of dynamic methylation sites on cryogenic electron microscopy structure of the human ribosome. SNORD78 guided site is shown in red; other dynamic sites in blue; rRNA and r-proteins in gray. Protein Data Bank code for the structure is 4UGO. Modification sites from 4UGO are reannotated to the rRNA sequence used for RiboMethSeq.

Furthermore, hierarchical clustering indicated that gain(1q) was also associated with specific snoRNA profiles. Indeed, comparing patients with gain(1q) to patients without, we identified 43 up- and 19 downregulated snoRNAs (Fig. 1A, B). The majority of upregulated snoRNAs in patients with gain(1q) were located in this region (*n* = 13/29 vs 30/596, Fisher’s exact test, *p* < 0.001, Fig. 1C).

Next, we took a closer look at snoRNAs on 1q as they are generally overexpressed. Among them, we noticed SNORD78, SNORD75 and SNORD47, all of which were overexpressed in gain(1q) patients with log2FC > 1 and were located on 1q25. To investigate whether snoRNAs in other regions on 1q or other chromosomes have similar features, we examined the distribution of all individual snoRNAs and mRNAs on each chromosome (Fig. 1D). Interestingly, snoRNAs located in other regions of 1q displayed neither significant upregulation nor gene-dosage effect. Notably, SNORD78 expression positively correlated with the copy number of 1q (Fig. 1E). RiboMethSeq revealed that the methylation level of SNORD78-guided ribosomal sites increased in a stepwise manner in patients without 1q aberration, gain(1q) up to the levels observed in patients with amp1q (Fig. 1F). Of note, the sites with increased 2′-O-methylation upon gain(1q) were located at the ribosome surface including the SNORD78-guided site 28S-4593 (Fig. 1G). Given that increases in ribosomal 2′-O-modification might increase pathogenetic potential in hematological malignancy [6], it is possible that SNORDS78 and rRNA methylation contribute to the poor prognosis of gain(1q). In line with our study, Krogh et al. observed an elevation of both SNORD78 expression level and methylation level in diffuse large B cell lymphoma (DLBCL), compared to reactive lymph node cells [10].

**Fig. 2:**
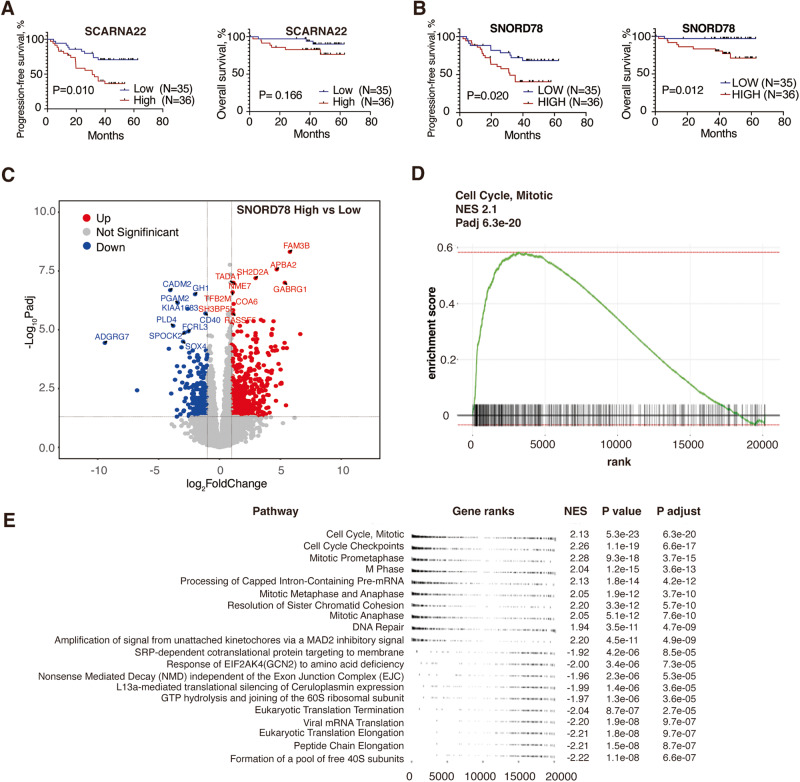
SNORD78 was upregulated in gain1q patients and associated with poor survival. **A** Kaplan–Meier curves of PFS and OS in patients with SCARNA22 high/low expression level. Higher expression level of SCARNA22 predicts poor PFS (31.06 months vs unreached, *p* = 0.007). **B** Kaplan–Meier curves of PFS and OS in patients with high/low SNORD78 expression. High expression of SNORD78 is associated with poor OS and PFS (*p* = 0.011, 0.019, respectively). **C** Volcano plot of differentially expressed genes (SNORD78 high vs low). **D** GSEA analysis of genes in patients with high expression of SNORD78. **E** Cell cycle pathways are activated in patients with high expression of SNORD78.

To determine the prognostic values of snoRNAs, we evaluated the outcome association of snoRNAs which were highly expressed in genetic high-risk diseases including t(4;14) and gain(1q). High expression of SCARNA22 was associated with shortened PFS (Fig. 2A, 31.06 months vs median not reached, *P* = 0.007; median OS not reached in both groups, *P* = 0.2). As expected, there was a trend that patients with amp(1q) showed worse PFS compared to patients without gain(1q), although in this cohort it did not reach statistical significance (Fig. S6). We also divided patients into 1q-snoRNAs high and low groups based on the sum normalized counts of all snoRNAs on chr1q. The cutoff point was the 50th percentile of snoRNA expression level. Similarly, patients with high 1q-snoRNAs tended to have inferior PFS (35.1 months vs median not reached, *P* = 0.096). Then we examined in detail the prognostic value of individual snoRNAs on 1q, particularly those in 1q25 region. Among 29 snoRNAs on 1q, SNORD78 was the only differentially expressed snoRNA associated with inferior OS and PFS (high vs low: median PFS 31.74 months vs median not reached, *P* = 0.019; median OS not reached in both groups, *P* = 0.011, Fig. 2B). In the multivariate COX analysis, SNORD78 was associated with PFS and OS with HR of 3.73 and 14.4 respectively, and corresponding p-values of 0.024 and 0.038 (Supplementary Table S2). We further analyzed the correlation between SNORD78 and factors such as. age, sex, LDH, ISS stage, but found no significant associations.

To further characterize MM with upregulation of SNORD78, we compared the bulk mRNA-Seq data between the SNORD78 *high* and *low* expression groups (50% as cutoff). In the SNORD78 *high* vs *low* group 537 protein-coding genes were upregulated, *e.g. SH2D2A* and *CDK1*, and 229 protein-coding genes were downregulated (Fig. 2C). Gene set enrichment analysis (GSEA) showed that cell cycle-related pathways were enriched in the SNORD78-high group (Fig. 2D, E). Several lines of evidence indicated that SNORD78 is highly expressed in multiple types of tumors and promotes tumor cell proliferation [11, 12]. Zheng et al. observed a marked upregulation in NSCLC tissues compared to normal tissues, coupled with worse overall survival [12]. Mechanistically, they observed that overexpression of SNORD78 promoted cell proliferation in vitro and SNORD78 depletion delayed in vivo tumorigenesis in nude mice. At the molecular level, the G0/G1 arrest markers, p21 and p16, were elevated and the proportion of G0/G1 cells increased consequently [12]. Those studies support our findings that SNORD78 and its mediated rRNA methylation might play an oncogenic role in MM.

Taken together, chromosomal aberrations consistently lead to altered snoRNA expression levels with concomitant changes of ribomethylomes in MM. These findings suggest that altered snoRNA expression may contribute to the adverse phenotypes observed in MM with common chromosomal abnormalities.

## Supplementary methods

### Sample collection

Overall, bone marrow samples were collected from 71 newly diagnosed multiple myeloma (NDMM), who received autologous stem cell transplantation after triplet or quadruplet induction and high-dose melphalan, and four relapsed or refractory multiple myeloma (RRMM) patients. Plasma cells were purified using CD138 microbeads (Miltenyi Biotech, Bergisch Gladbach, Germany). Written informed consent was obtained prior to sampling in accordance with the Declaration of Helsinki. This study was approved by the Institutional Ethics Committee. Three specimens of CD19+ B cells from healthy donor peripheral blood were sorted as control. Fluorescence in situ hybridization (FISH) analysis was performed for the detection of and the determination of copy number changes as recently described [13]. Cases with at least two trisomies of odd-numbered chromosomes were classified as hyperdiploid.

### Bulk mRNA sequencing

Total RNA was isolated with the AllPrep DNA/RNA/miRNA Universal Kit (Cat. No.: 80224, Qiagen, Hilden, Germany) according to the manufacturer’s manual. The mRNA NGS libraries were prepared using the TruSeq stranded mRNA library kit (Cat. No.: 20020595, Illumina, San Diego, CA, USA). Sequencing of mRNA was performed on an Illumina NovaSeq 6000 PE 100 S1 platform. Paired-end RNA-seq reads were mapped to the hg19 reference genome using STAR. Gene expressions were quantified using featureCounts.

### snoRNA sequencing

Libraries were constructed using the NEBNext multiplex small RNA library (Cat. No.: E7300S, New England Biolabs, Frankfurt, Germany) according to the instructions of the manufacturer. The barcoded libraries were size selected for inserts ranging from 40 to 200 bp using AMPure XP Beads (Beckman Coulter). Sequencing of single-end 75 bp (75SE) was performed with an Illumina Nextseq500.

### RiboMeth sequencing

Twenty-six NDMM samples with ≥100 ng RNA were analyzed by RiboMeth sequencing. Briefly, 100 ng total RNA was hydrolyzed in an alkaline buffer (50 mmol/L bicarbonate, pH 9.2) at 95 °C for 12 min to achieve an average fragment size of about 30 nucleotides. The RNA fragments were purified using Oligo Clean&Concentrator Kit (Cat. No.: D4062, Zymo, Freiburg, Germany) according to the manual and then proceeded to end repair with 5 U of Antarctic Phosphatase (NEB) for 30 min at 37 °C for 3′-end dephosphorylated. After heat inactivation of the phosphatase, the 5′-end of RNA fragments were phosphorylated using T4 PNK and 1 mM ATP for 1 h at 37 °C. Libraries were prepared using the NEBNext Small RNA library prep kit, followed by single-end sequencing on an Illumina NextSeq 500. Reads trimming was performed with cutadapt to remove the adapter sequence, and reads below 15 nucleotides were discarded. The filtered reads were mapped to rDNA sequences with the bowtie2 reference sequence file containing rDNA sequences of U18, U28, and U5.8S. Methylation was calculated by ScoreC based on the reads on 5′-end of the fragment as described before [14]. Annotation of modification sites, as well as rRNA sequence, was downloaded from https://www-snorna.biotoul.fr.

### snoRNAseq data analysis

Small-RNA-seq reads were trimmed using the cutadapt software (version 2.5). Next, trimmed reads were aligned subsequently to respective genomes (rRNA: https://www.ncbi.nlm.nih.gov/nuccore/555853, tRNA: http://gtrnadb.ucsc.edu/genomes/eukaryota/Hsapi19/hg19-tRNAs.fa, human: hg38) with bowtie2 (version 2.2.5). Mapped reads overlapping published snoRNAome regions (PMID: 27174936) were extracted [15], assigned by overlap and counted using the R environment for statistical computing including bioconductor packages GenomicAlignments and GenomicRanges. SnoRNA mapping profiles were manually inspected in order to ensure that we focus on high confidence full length snoRNA spanning reads with respect to their mapping position in the annotated snoRNA region. In case we found massive amount of reads which are either starting outside the expected 5′-region or inside the actual snoRNA region suggesting being a result of a fragmentation process, we established custom scripts to separate full length snoRNA reads from other. Read counts of other (not full length snoRNA) are provided in the count table as well. The normalized count matrix was generated using DESeq2 (version 1.34.0). SnoRNAs differentially expressed in MM samples with different clinical characters were identified with the threshold fold change >1, FDR <0.05.

### Statistical methods

Differentially expressed snoRNAs identified from DESeq2 (padj < 0.05) were plotted in volcano plots. We defined upregulation as a log2 fold change (log_2_FC) > 0 with an adjusted *P* value (padj) <0.05. The heatmap was generated using the R pheatmap package. The Kaplan-Meier method was used for survival analyses. Progression-free survival (PFS) time was calculated from baseline to relapse or death from any cause, whichever occurred first. Curves were compared by univariate (log-rank) analysis using GraphPad Prism version 9.5. A *p*-value less than 5% was considered statistically significant. No correction was performed for multiple testing and all analyses should be deemed exploratory.

### Supplementary information

Supplementary Figures and Tables

## Data Availability

Sequencing data are available on GSE237999.
