# Supplementary material for: The landscape of small nucleolar RNA expression in multiple myeloma is determined by cytogenetic alterations
Source: Leukemia. 2023 Oct 16;37(12):2526–31. doi: 10.1038/s41375-023-02060-2 (PMC10681893; doi:10.1038/s41375-023-02060-2)
Supplement: Supplementary file 1 — Supplementary Figures and Tables [file 41375_2023_2060_MOESM1_ESM.pdf]

Supplementary Figures

Figure S1: <u>Patterns of snoRNA expression and ribosomal RNA methylation in multiple myeloma</u>

A. Hierarchical clustering of the patient specimens with the top 100 most variably expressed snoRNAs (patients in columns, snoRNAs in rows). Unsupervised analyses identified chromosomal imbalances as a main feature of various clusters: color purple (cluster1: Hyperdiploid patients), color blue (cluster2: IgH cases), color green (cluster3: patients with gain1q), as shown in boxes.
B. Overview of ribosomal rRNA 2'-O-Methylation patterns in primary multiple myeloma samples. The rRNA sites with dynamic changes between myeloma specimens are indicated in red whereas the blue boxes indicate static methylation levels among patients.

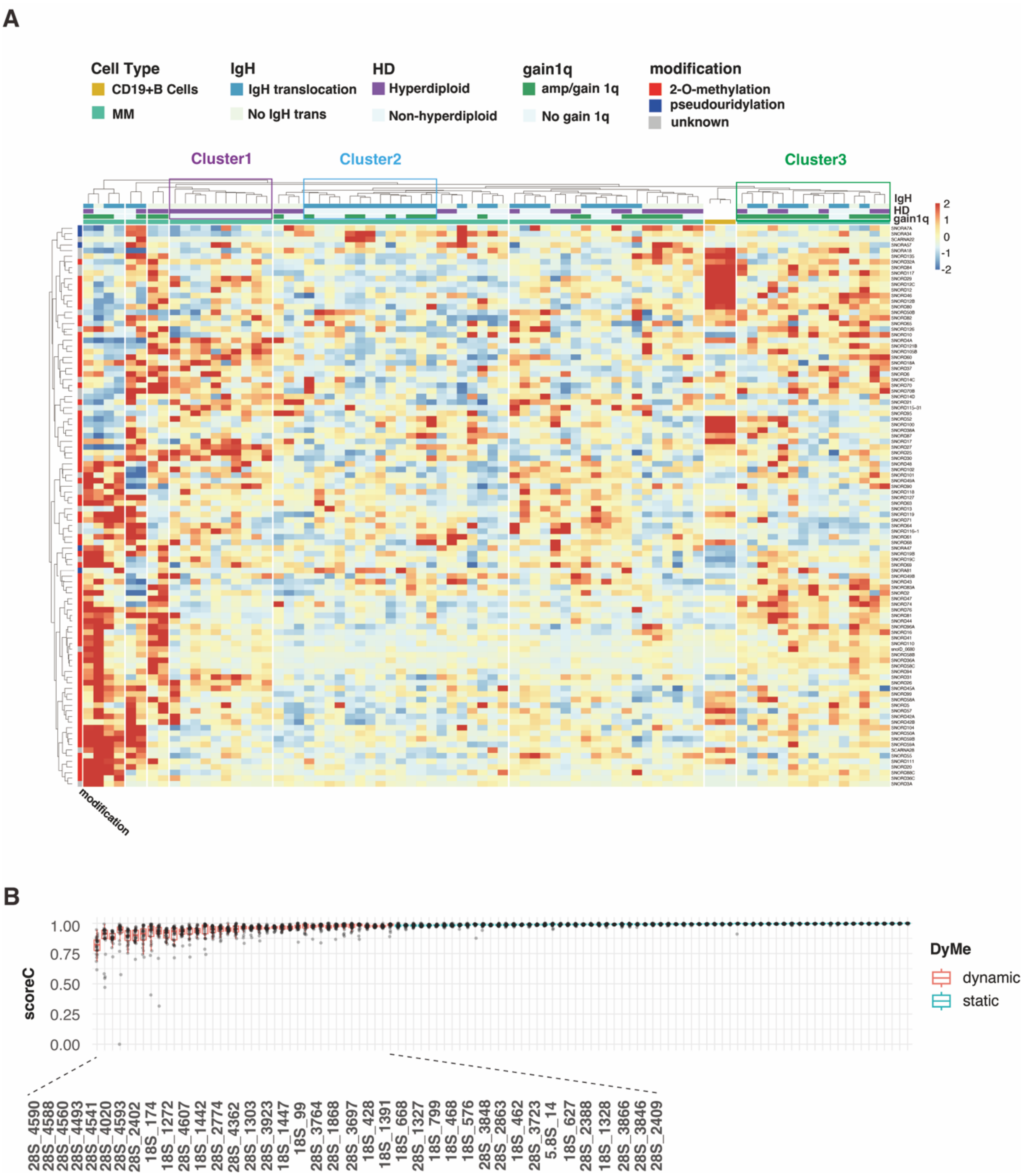
A
Cell Type
CD19+B Cells
MM
IgH
IgH translocation
No IgH trans
HD
Hyperdiploid
Non-hyperdiploid
gain1q
amp/gain 1q
No gain 1q
modification
2-O-methylation
pseudouridylation
unknown
Cluster1
Cluster2
Cluster3
IgH
HD
gain1q
modification
B
scoreC
1.00
0.75
0.50
0.25
0.00
DyMe
dynamic
static
28S_4590
28S_4588
28S_4560
28S_4493
28S_4541
28S_4020
28S_4593
28S_2402
18S_174
18S_1272
28S_4607
18S_1442
28S_2774
28S_4362
28S_1303
28S_3923
18S_1447
18S_99
28S_3764
28S_1868
28S_3697
18S_428
18S_1391
18S_668
28S_1327
18S_799
18S_468
18S_576
28S_3848
28S_2863
18S_462
28S_3723
5.8S_14
18S_627
28S_2388
18S_1328
28S_3866
28S_3846
28S_2409

Figure S2: <u>Differential expression of snoRNAs in genetic subsets of multiple myeloma</u>

A. Volcano plot of differentially expressed snoRNAs in Hyperdiploid vs non-HD patients. Scattered points represented individual snoRNA: *red*-snoRNAs on chr15, *grey*-snoRNAs outside chr15. Red solid lines represented *Padj* < 0.05.

B. Heatmap of differentially expressed snoRNAs (padj < 0.05) in hyperdiploid (HD) vs non-HD patients

C. Upregulated snoRNAs of HD patients are mainly located on chr15. Each dot is a single snoRNA, mRNA, respectively.

D. Box plot of SNORD115 family expression level in hyperdiploid (HD) patients without gain1q, vs HD patients with gain1q.

E. Volcano plot of differentially expressed snoRNAs in t(4;14) vs non-t(4;14) patients.

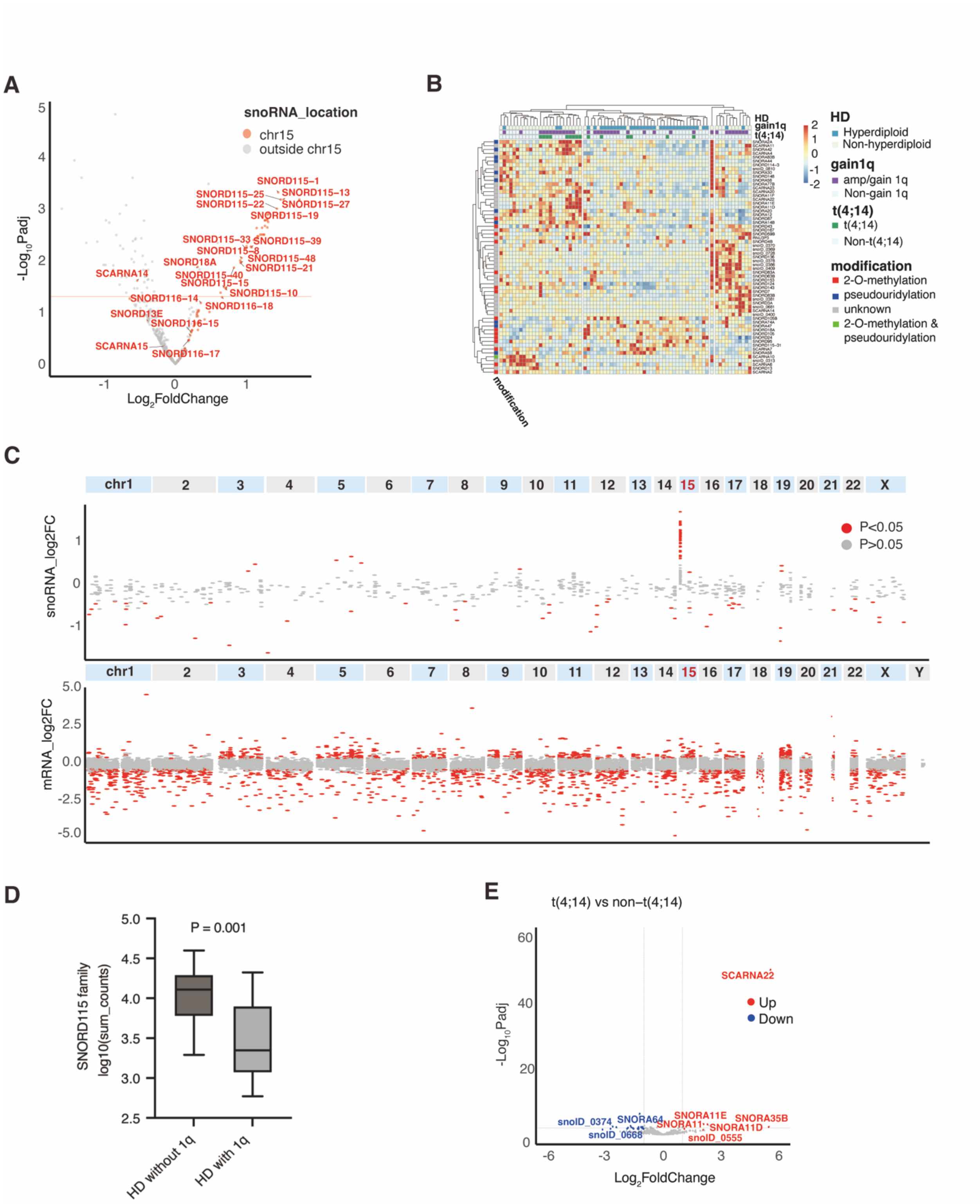

A
snoRNA_location
chr15
outside chr15
-Log10Padj
Log2FoldChange
SNORD115-1
SNORD115-25
SNORD115-13
SNORD115-22
SNORD115-27
SNORD115-19
SNORD115-33
SNORD115-39
SNORD115-8
SNORD115-48
SNORD18A
SNORD115-21
SCARNA14
SNORD115-40
SNORD115-15
SNORD115-10
SNORD116-14
SNORD116-18
SNORD13E
SNORD116-15
SCARNA15
SNORD116-17
B
HD
gain1q
t(4;14)
Hyperdiploid
Non-hyperdiploid
amp/gain 1q
Non-gain 1q
Non-t(4;14)
modification
2-O-methylation
pseudouridylation
unknown
2-O-methylation & pseudouridylation
C
chr1
snoRNA_log2FC
P<0.05
P>0.05
mRNA_log2FC
D
P = 0.001
SNORD115 family log10(sum_counts)
HD without 1q
HD with 1q
E
t(4;14) vs non-t(4;14)
-Log10Padj
Log2FoldChange
SCARNA22
Up
Down
snoID_0374
SNORA64
snoID_0668
SNORA11E
SNORA11
SNORA35B
SNORA11D
snoID_0555

Figure S3: **<u>SNORD115 family expression level in different subgroups</u>**

A. Box plot of SNORD115 family expression level in patients with <3 copies, 3 copies, >3 copies of chr 15. NS: No significance

B. Box plot of SNORD115 family expression level in HD patients with trisomy15, HD patients without trisomy15, Non-HD patients.

C. Box plot of SNORD115 family expression level in patients with <3 copies, 3 copies, >3 copies of chr 5.

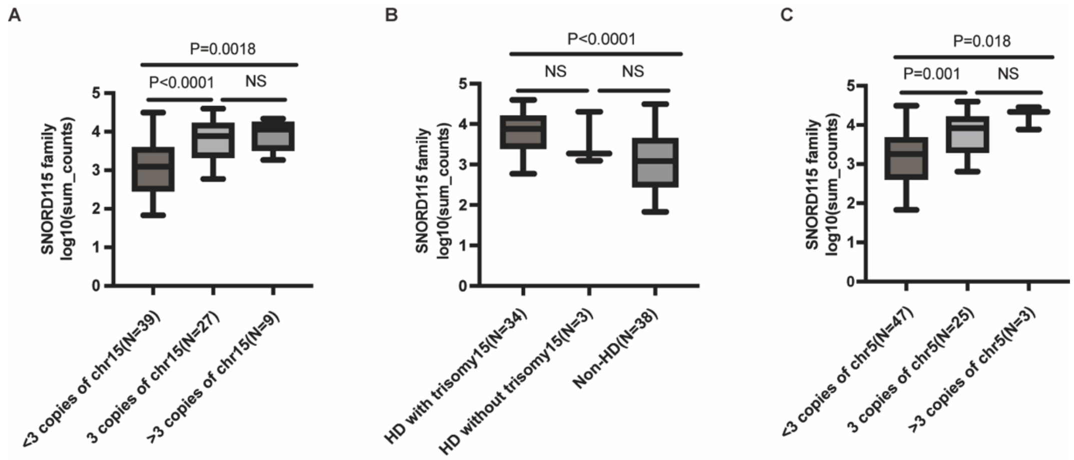

Figure S4 <u>Heatmap of differentially expressed snoRNAs in t(4;14) vs non-t(4;14) patients.</u>

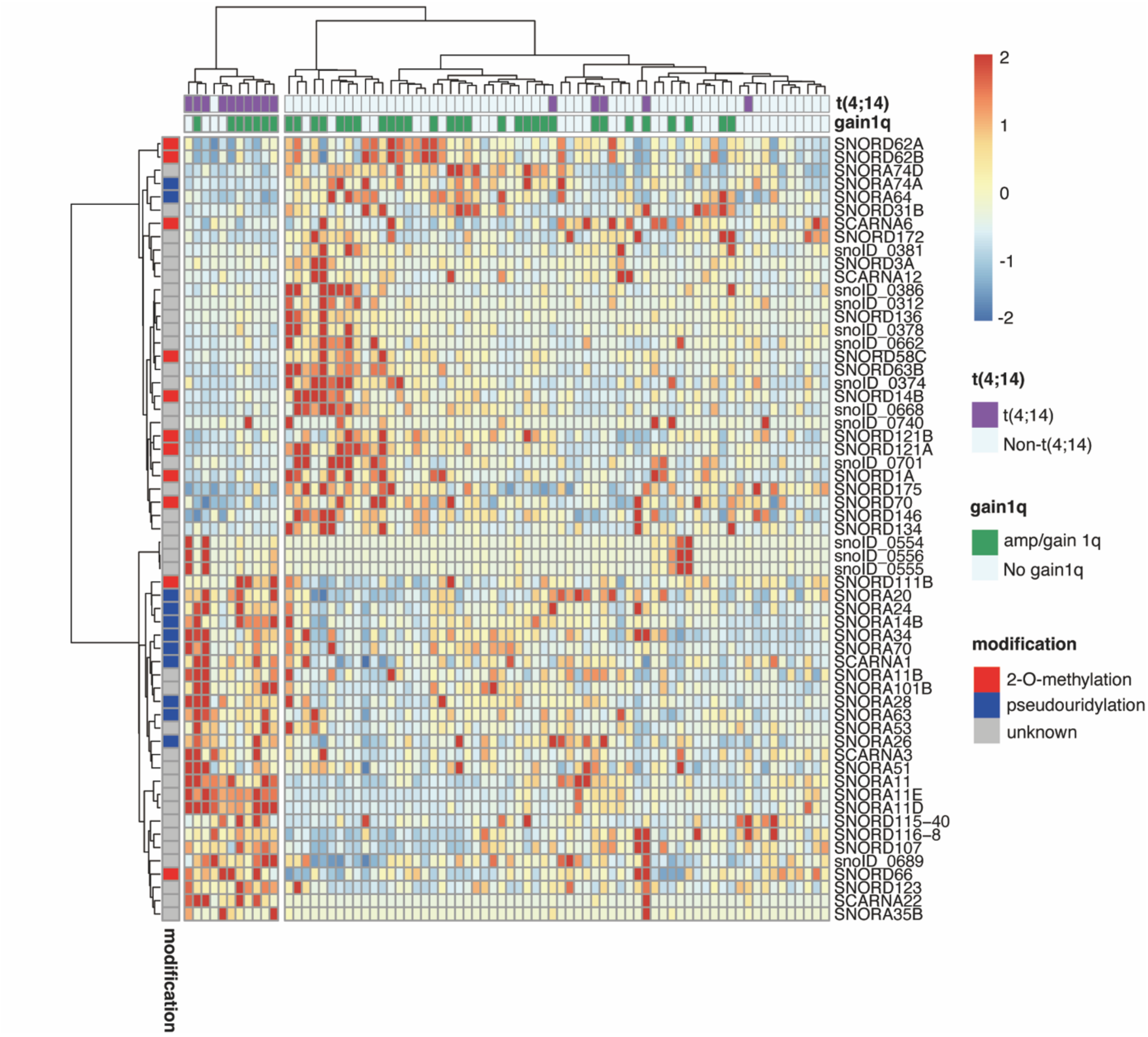

Figure S5 **<u>Heatmap of differentially expressed snoRNAs in t(11;14) vs non-t(11;14) patients.</u>**

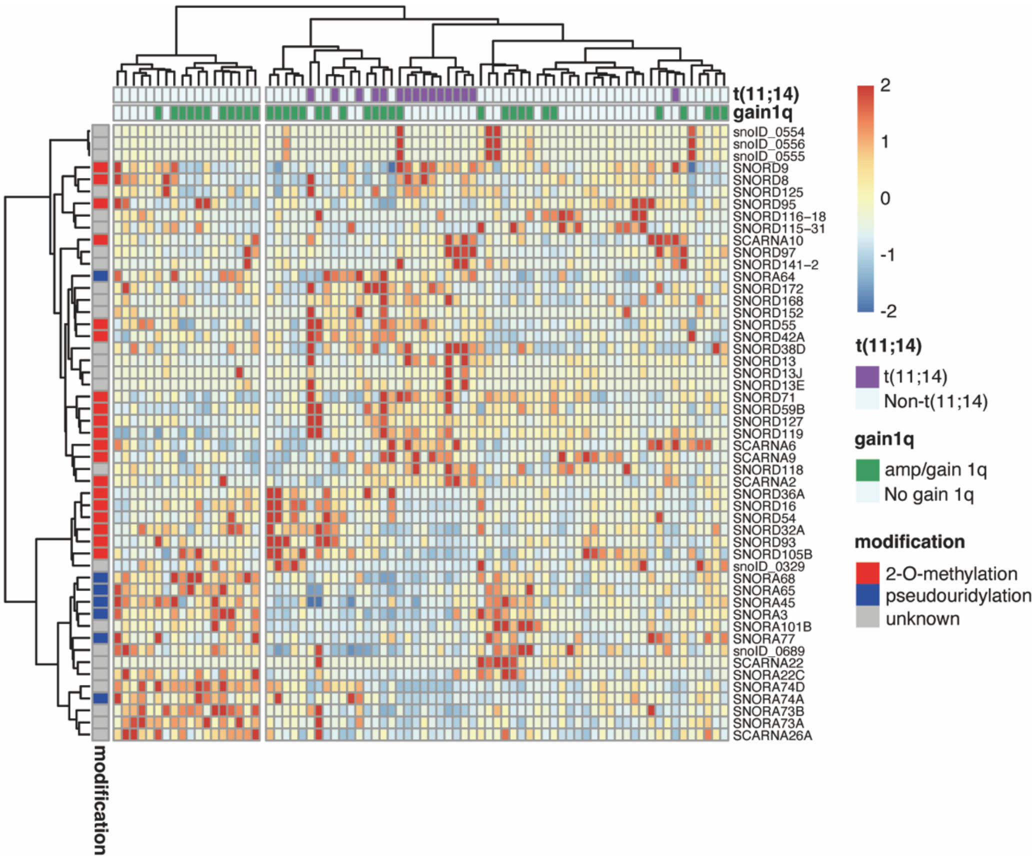

Figure S6 <u>Kaplan-Meier curves of *gain1q*, *amp* and *non-gain1q* patients</u>

A. PFS of *gain1q*, *amp* and *non-gain1q* patients.

B. OS of *gain1q*, *amp* and *non-gain1q* patients.

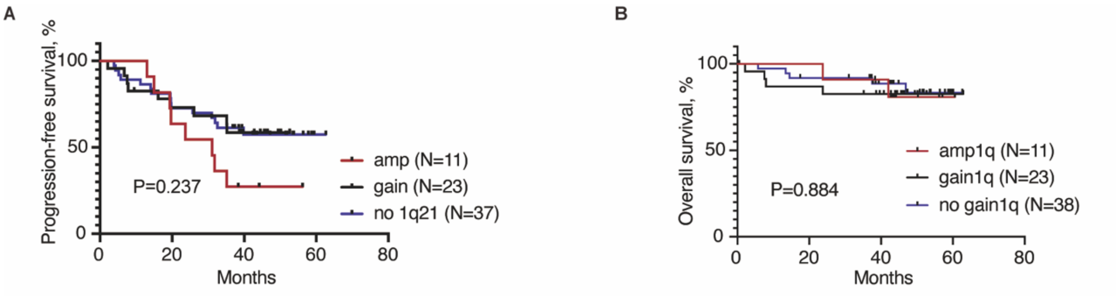

Supplementary Tables

Table S1. Clinical characteristics of myeloma patients

		**Patients (N=75)**
Age (Median, range)		58 (42-70)
Sex	Male	42 (56.0%)
	Female	33 (44.0%)
WHO PS	0	36 (48.0%)
	1	29 (38.7%)
	2	9 (12.0%)
	3+	1 (1.3%)
ISS Stage	I	32 (42.6%)
	II	26 (34.7%)
	III	17 (22.7%)
Hyperdiploid(HD)	HD	37 (49.3%)
	Non-HD	38 (50.7%)
1q21 gain	1q21 gain	24 (32.0%)

	1q21 amp	12 (16.0%)
	No 1q21 gain	39 (52.0%)
Del 17p	Del 17p	11 (14.7%)
	No del17p	64 (85.3%)
LDH elevation	Normal LDH	13 (17.3%)
	LDH elevation	62 (82.7%)
IgH translocation	t(4;14)	15 (20.0%)
	t(6;14)	3 (6.7%)
	t(11;14)	16 (21.3%)
	t(14;16)	5 (4.0%)
	No IgH translocation	36 (48.0%)

Table S2. Multivariate COX model for Progression Free Survival and Overall Survival

Variable	Multivariate COX for **PFS**			Multivariate COX for **OS**		
	HR	95% CI for HR[a]	P value	HR	95% CI for HR[a]	P value
Age(years)	0.99	0.93-1.04	0.632	0.99	0.89-1.10	0.826
Male vs Female	0.64	0.30-1.37	0.254	0.31	0.07-1.28	0.105
ISS Stage II vs I	4.16	1.58-10.9	0.004*	2.26	0.37-13.7	0.374
ISS Stage III vs I	2.94	1.04-8.29	0.042*	4.50	0.70-29.8	0.119
LDH evelation vs normal	1.11	0.42-2.93	0.826	4.32	0.99-18.8	0.051
del17p	0.396	0.10-1.55	0.183	1.61	0.32-8.05	0.563
t(4;14)	0.828	0.28-2.41	0.729	2.12	0.17-26.4	0.561
amp/gain1q	0.39	0.13-1.18	0.096	0.37	0.08-1.83	0.223
SNORD78 expression high vs low	3.73	1.19-11.7	0.024*	14.4	1.17-179	0.038*

a. HR: hazard ratio; CI: confidence internal
